# Effects of turmeric (*Curcuma longa*) and vitamin E on histopathological lesions induced in bursa of Fabricius of broiler chicks by salinomycin 

**Published:** 2017-09-15

**Authors:** Reza Sayrafi, Navideh Mirzakhani, Reza Mobaseri

**Affiliations:** 1 Department of Pathobiology, Faculty of Veterinary Medicine, Amol University of Special Modern Technologies, Amol, Iran;; 2 Department of Pathology, Faculty of Veterinary Medicine, Urmia University, Urmia, Iran;; 3 DVM Graduated, Faculty of Veterinary Medicine, University of Shiraz, Shiraz, Iran.

**Keywords:** Bursa of Fabricius, Chick, Salinomycin, Turmeric, Vitamin E

## Abstract

The aim of this study was to evaluate the protective effects of the turmeric in comparison to vitamin E on bursal damages induced by salinomycin in broiler chickens. In this study, forty one day-old broiler chicks were randomly divided into four treatment groups: 1- basal diet as control, 2- basal diet plus salinomycin, 3- basal diet plus salinomycin (SLM) and vitamin E (Vit. E) and 4- basal diet plus salinomycin and turmeric powder. The chicks were treated for two weeks. At the end of the experiment, the bursal tissues were removed and fixed in 10% formalin solution. Tissue sections were stained with hematoxylin and eosin stain for histopathological studies. Light microscopic observations showed that, SLM diminished cortex thickness of bursal tissue, enhanced its medulla zone and caused severe lymphocytic necrosis. In addition, SLM led to fibrosis of interstitium along with sever edema of medulla zone in the bursal tissue of the chicken. Administration of Vit. E and TP significantly inhibited the SLM-induced derangements and comparing the Vit. E and TP showed no significant differences. The results of this study indicated that the turmeric may protect bursa of Fabricius against toxicity induced by salinomycin in chicks.

## Introduction

In birds, the bursa of Fabricius is a lymphoid organ that is the thymus counterpart for B cells. It is believed that the bursa is a primary lymphatic organ for B cell differentiation.^1^

The ionophores are a group of antibiotics, which have been used commercially as anticoccidial agents in broiler chickens to improve the efficiency of feed utilization.^2^ Ionophores modify the cell membrane permeability and facilitate the influx of ions, but may cause severe functional and morphological disturbances in cells. These compounds could induce toxic syndromes in the case of overdose and misuse.^3^ The risk of possible intoxication is not only with regard to overdosing due to a narrow range of safety, but some of them are highly toxic to some animal species and special age groups of animals. So that, it is recommended that their safe application be justified entirely.^4^^-^^6^ Ionophore toxicity could be probably due to the free radical-mediated oxidative damage and such damage can be prevented by the supplementation of antioxidants in the feed.^7^^,^^8^

Vitamin E (Vit. E), a fat-soluble vitamin of plant origin, is the most important lipid-soluble antioxidant. Humans and animals are unable to synthesize Vit. E within their body and must obtain it from plant sources and its concentration is usually reduced below the normal under stress conditions.^9^ It is suggested that antioxidants’ deficiencies, particularly Vit. E could play a role in the development of the disease.^10^^,^^11^ This vitamin functions in biological systems primarily as a protective agent against free radicals.^12^ The immunoregulatory effects of dietary Vit. E on humoral and cell-mediated immunities are well established and its beneficial impact on the overall immunocompetence of broilers has been reported.^13^^,^^14^

Several studies have declared a beneficial role of the rhizome of turmeric (*Curcuma longa*) in terms of antioxidant,^15^ antimicrobial,^16^ antitumourgenic,^17^ and tissue-protective properties.^18^ The major pigment in turmeric is curcumin, which is a major active component of turmeric. The salient feature of curcumin is to exhibit strong antioxidant activity, which is comparable to Vitamins E and C.^19^ However, no study has so far investigated the protective effects of turmeric powder (TP) and Vit. E on bursal of Fabricius damage induced by salinomycin (SLM) in broilers. In this study, we assessed the protective effects of the turmeric in comparison to Vit. E against bursal damage induced by SLM in broilers.

## Materials and Methods

**Birds and treatments.** Forty one day-old broiler chicks (Cobb strain) were obtained from a commercial hatchery. The birds were randomly divided into four groups. In each group, 10 chicks were included for 35 days. The experimental groups were: 1) basal diet as control, 2) basal diet plus SLM, 3) basal diet plus SLM and Vit. E and 4) basal diet plus SLM and TP.

**Feeding and feed additives.** Feeding of the chicks was based on starter, grower and finisher rations. The ration was the same for all groups and was based on corn and soybean. In treatment groups, SLM, Vit. E and TP were added to the basal diet at the doses of 300, 100 and 1000 mg kg^-1 ^body weight per day, respectively from day 22 to day 35 (for 14 days).

**Histopathological examination.** At the end of the experiment, five chicks were selected from each group. For histomorphometric analysis, samples from bursal tissues were transected and fixed in 10% formalin. Tissues were dehydrated by transferring through a series of alcohols with increasing concentrations, placed into xylol and embedded in paraffin. From each specimen, eight sections (5 to 6 µm) from bursal tissues were prepared and stained with hematoxylin and eosin (H & E) for light microscopic observations. The evaluation of the sections were based on the severity of pathological changes on a scale from normal (0) to severe (3) changes.^20^

**Statistical analysis.** Statistical analysis was performed using SPSS software (version 21; SPSS Inc., Chicago, USA). The results were subjected to one-way analysis of variance followed by Duncan’s multiple-range test. Significance at *p* < 0.05 has been given receptive in all tests.

## Results

**Fig. 1:**
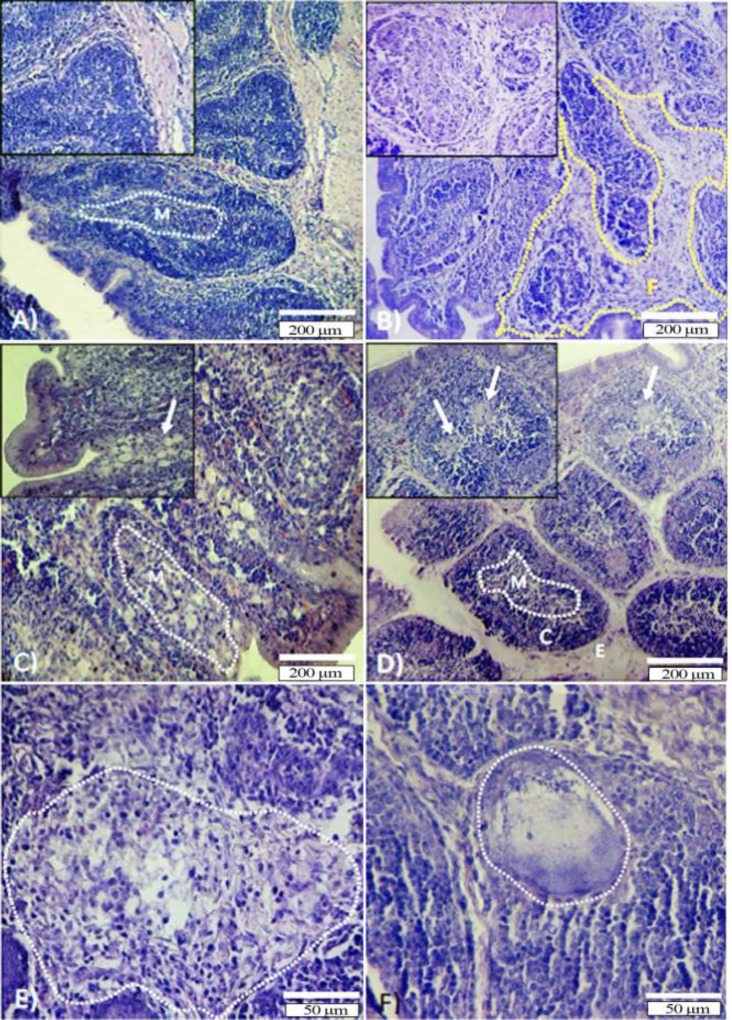
Cross sections from bursa of Fabricius of examined chickens. **A**) Control group; the normal structure of bursa in control chicken. Cortex (C) and medulla (M) of follicles are rich in lymphocytes. See normal interestitium with no edema; **B**) SLM-treated group; Marked lymphocytic depletion is presented in both cortex and medulla. Severe follicular atrophy associated with fibrotic (F) interfollicular interestitium (dotted line) is presented in the bursa. Note the reticular cells proliferation in insert section; **C**) SLM + TP group; See moderate depletion of the lymphocytes in the medulla and cortex. Note higher magnification for lymphocytic depletion in the medulla (arrow in inset); **D**) SLM + Vit. E group; mild depletion with reticular cells proliferation in medulla. Note regenerated lymphocytes in the cortex and medulla. High magnification for proliferation of reticular cells is showed in insert section (arrows); **E**) High magnification from completely atrophied follicle with no clear boundary between the cortex and medulla from the SLM-treated group; **F**) Large cystic cavitation in a bursal follicle of the SLM-treated group containing faint eosinophilic necrotic debris, which is free from lymphocyte (H & E staining

The cortex of the lymphoid follicles of the bursa of Fabricius was narrow and the medulla was markedly enlarged in SLM-treated birds. Observations revealed the bursa of SLM-treated birds with depleted lymphocytes and lymphocytes degeneration, particularly in the medulla of lymphoid follicles as well as the cortex. The severe follicular atrophy and enlarged fibrotic interstitium associated with severe edema were observed in the SLM-treated group (Fig. 1). The percentage of bursal follicles with type 1, 2 and 3 depletions was higher significantly (*p *<0.05) in the SLM-treated group than the TP and Vit. E administrated group (Table 1). Moreover, the bursal follicles were seen with large cystic cavitations, containing faint eosinophilic necrotic debris in the SLM-treated group (Fig. 1).

In the Vit. E-treated group, the thickness of cortex and medulla increased, in which the boundary between cortex and medulla was mingled (Fig. 1) and the follicular diameter increased (Table 2). The lymphocytic compaction was increased in cortex and medulla of the follicles. Mild reticular cells proliferation was observed in this group and the interstitial edema was limited (Fig. 1). 

The bursa of chickens in the TP-treated group showed moderate lymphocytic depletion (Table 1) and reticular cells proliferation. The interstitial tissue exhibited reduced edema and fibrosis in comparison to the SLM-treated group (Fig. 1). Similar to Vit. E-treated group the follicular diameter elevated in the TP-treated group, while there were no statistically significant differences between two treatment groups (Table 2). The data for lymphocytic depletion and follicular diameter are presented in Tables 1 and 2.

**Table 1:** Lymphocytic depletion and percentage of follicles with lymphocytic depletion in bursa of Fabricius of salinomycin (SLM), turmeric (TP) and vitamin E (Vit. E) administered chickens. All parametric data are presented in mean ± SD

**Groups**	**Lymphocytic depletion (1/2/3)**	**Depletion (1)**	**Depletion (2)**	**Depletion (3)**
**Control**	0	0	0	0
**SLM-treated**	3	23.13 ± 4.38^a^	12.38 ± 1.61^a^	8.62 ± 1.02^a^
**SLM + Vit. E-treated**	1	12.33 ± 1.53^b^	5.27 ± 1.08^b^	0.96 ± 0.01^c^
**SLM + TP-treated**	2	15.08 ± 2.64^b^	8.63 ± 1.11^b^	2.30 ± 1.27^b^

abc Superscript letters indicate significant differences among the groups in the same column at *p* <0.05. Note: 0: No lesion; 1: Mild depletion; 2: Moderate depletion; and 3: Severe depletion.

**Table 2:** Morphometric data for follicular diameter in bursa of Fabricius of salinomycin (SLM), turmeric (TP) and vitamin E (Vit. E) administered chickens. All data are presented in mean ± SD

**Groups**	**Follicular diameter (µm)**
**Control**	237.44 ± 41.40^a^
**SLM-treated **	174.68 ± 16.28^b^
**SLM + Vit. E-treated**	231.50 ± 14.54^a^
**SLM + TP-treated**	225.36 ± 31.07^a^

ab superscript letters indicate significant differences among groups at* p* < 0.05.

## Discussion

Accidental or experimental ionophorus intoxication has been reported in a number of species, such as chickens, quails, turkeys, rabbits and horses.^21^^-^^25^ Several medicinal and pharmacological properties such as antioxidant,^15^ anti-inflammatory,^26^ tissue-protective^18^ and immunostimulant^27^ activities of turmeric have been reported. Turmeric is a plant with a very long history of medicinal application which several studies have shown its safety and non-toxicity for animals and humans.^28^ The results of our study showed that treatment with turmeric had protective and therapeutic effects on the SLM toxicity in the bursa of Fabricius of chickens.

In this study, statistical analysis of data revealed severe depletion of lymphocytic cells in the SLM group. This observation is in agreement with the findings obtained by Hussein *et al.*, who reported the toxic dose of SLM leads to depletion of follicles’ central area in bursa of Fabricius.^23^ In Vit. E-treated group, just a mild lymphocytic depletion observed in the bursa of chicks. These findings were in agreement with Sodhi *et al.*, who opined that dietary supplementation with Vit. E and selenium possess influence on the immunological traits in broilers.^29^ Tayeb and Qader suggested that the addition of Vit. E and selenium in the broiler’s diet lead to improve the immunity in broiler by increasing the number of lymphocytes.^30^ Vit. E is known for its antioxidant property, which protects phospholipids of the cell membrane against free radical damage. The protective effect of Vit. E against the SLM damage could be attributed to the role of Vit. E as an antioxidant which protects the biological membranes from oxidative damage.^31^ In the TP-treated group, moderate lymphocytic depletion in the bursa was observed and the overall tissue structure was notably improved in the terms of depletion and lymphocytic necrosis compared with SLM- treated group. A recent study showed that dietary supplementation with turmeric or curcumin enhances immunity and induces protective immunity and antioxidative activity against free radicals.^32^ In agreement with our result, Madhavi and Saraswathi observed therapeutic effect of tumeric against the chlorpyrifos (an organophosphate insecticide) toxicity in mice,^33^ but no reports are available on the protective effect of tumeric in alleviating the immunotoxic effects of ionophores in bursa of Fabricius in chickens.

In the present study, follicular diameter in the SLM group decreased compared with the control group and showed severe follicular atrophy. Similar to Vit. E-treated group the follicular diameter elevated in the TP-treated group, while there were no statistically significant differences between these treatment groups with the control group. Atrophy of the bursal tissue as a result of lymphocytic depletion is produced by various reasons consist of consuming immunosuppressive drugs, encountering nutritional diseases (vitamin deficiency) and viral or bacterial diseases.^34^ These findings are in agreement with the findings of Shalaby *et al. *and Hussein *et al.*, who reported the immunosuppressive effect of SLM toxicosis by lowering antibody titers in Newcastle disease as well as reducing the relative weight of lymphoid organs.^23^^,^^35^ In a study, the histopathological evaluation revealed an immunosuppressive effect of malathion toxicity on the bursa of Fabricius and bursal atrophy in quails.^36^ Sodhi *et al.* reported a prominent decrease in the weight of the bursa of the chicks in the group treated with malathion compared to the control group. There was a significant elevation in weight of the bursa of the chicks which received diet supplemented with vitamin E and selenium compared to those in the group given malathion alone.^29^ In addition, Narendra reported that turmeric could reduce the damages induced by experimental salt toxicity in cockerel and increased the weight of bursa of Fabricius and spleen.^37^

 In this study, the bursa of the SLM group showed lymphocytes degeneration, large cycstic cavitations containing faint eosinophilic necrotic debris and enlarged fibrotic interstitium associated with severe edema. In agreement with our study, Hussein *et al.* described the microscopical lesions of the bursa of Fabricius due to SLM toxicosis in chickens as the presence of degenerative changes in the epithelial cells lining the mucosal layer along with depletion of the central portion of the follicles.^23^ The decrease in the follicular size in our investigation may be attributed to degenerative changes in the bursa of Fabricius. Several studies have reported that antioxidant agents such as Vit. E alleviate various toxicity.^29^^,^^38^ Turmeric’s tissue protective effect is mainly due to it’s antioxidants activity, which has been suggested as a possible mechanism of action of turmeric against cellular toxicity.^39^ Earlier reports suggested that total antioxidant activity and superoxide dismutase (SOD) and catalase (CAT) concentrations improved by the addition of turmeric in the broiler chicks’ diet.^40^^,^^41^ Also, other experimental studies have shown that curcumin has a strong antioxidant action in several vital organs including the liver, kidneys and heart and it has a free radical scavenging activity.^19,41,42^ Moreover, Curcumin is known to increase the antioxidant potential especially through SOD which could be due to the increased expression of SOD gene in the chickens fed turmeric.^43^

In this study, the severity of lesions in the bursa of Fabricius was reduced after addition of Vit. E and TP; thus it can be concluded that these additives may have a considerable effect in reducing SLM toxicity in the bursa of Fabricius.
